# State of Lunacy in England
*Seventh Annual Report of the Commissioners in Lunacy to the Lord Chancellor. 30th June, 1852. Pursuant to the Act 8 and 9 Vict., s. 88. (Ordered by the House of Commons to be printed, 5th April, 1853.)


**Published:** 1853-10-01

**Authors:** 


// THE JOURNAL

OF

PSYCHOLOGICAL MEDICINE

AND

MENTAL PATHOLOGY.

OCTOBER 1, 1853.

### Art. I.—STATE OF LUNACY IN ENGLAND.*

The Seventh Annual Report of the Commissioners in Lunacy has just been issued to the public. Before proceeding to analyse its contents, we feel it our duty to refer briefly to a grievance connected with its publication to which our attention has been specially called. We allude to the unreasonable delay which uniformly takes place in the publication of these annual reports, which seem to lag habitually something like a year and a half behind the time to which they relate. It is clear, that all public, official, or parliamentary reports should be brought down as close as practicable to the period of publication; they should not be a record of bygone, but of *existing* facts, otherwise they possess only a retrospective and not a present interest. The Seventh Annual Report of the commissioners in Lunacy is open to this objection. It extends from June 30th, 1851, to June 30th, 1852, and has not been published until the August of 1853! By the 88th section of the Act 8 and 9 Vict. cap. 100, it is enacted that the commissioners shall, at the expiration of every six calendar months, report to the Lord Chancellor the number of visits which they have made, the number of patients whom they may have seen, and the number of miles they have travelled during such months; which mileage would appear curious were it not intended as a criterion of the expenses thereby incurred. Furthermore, they are required in the month of June in every year to make a report to the

* Seventh Annual Report of the Commissioners in Lunacy to the Lord Chancellor. 30th June, 1852. Pursuant to the Act 8 and 9 Vict., s. 88. (Ordered by the House of Commons to be printed, 5th April, 1853.)

Lord Chancellor of the state and condition of the several houses, hospitals, asylums, and other places visited by them—the care taken of patients generally, and such other particulars as may appear to them deserving of notice. All this is unquestionably highly proper and important; but unhappily the same section of the Act provides that this report shall be laid before parliament within twenty-one days next after the commencement of every session; which probably may account in a great measure for the delay complained of in its publication. Undoubtedly it is a grievance. Here we have, in the August of 1853, the last annual report of the commissioners, which only brings down the admissions of the insane into asylums and licensed houses to the 1st January, 1852. Here we have a tabular view of the number of patients, private and pauper, found lunatic by inquisition, and criminal, confined in these metropolitan and provincial establishments; and yet these tables leave us so much in arrear that they are for our present information perfectly useless. The appointments, too, of superintendents and medical officers in these institutions, have, during the interval of publication, undergone material changes; yet this report, which was ordered by the House of Commons to be printed on the 5th April, 1853, retains the names of Mr. Eccleston, as superintendent of Rainhill, Dr. Conolly, visiting physician at Hanwell, and Dr. Bushnan's appears as if just gazetted for the "appointment" of medical superintendent at Wyke House. It is true that eighteen months or two years ago, such appointments were held; but these, and many others specified in the report before us, have for some time ceased to exist. But this is not all. The comments and censures which the commissioners may have thought proper to pass upon the condition and management of certain asylums *nearly two years ago*, are *now* for the first time published to the world, although sufficient time has elapsed for the removal of the causes which suggested their animadversions. It is only fair, therefore, to presume that many of the suggestions in the report before us, which were made by the commissioners so far back, have, before its publication, in most instances, been attended to; but, however promptly or efficiently the superintendents in charge of such asylums may have responded to the wishes of the commissioners, they must still remain under the public ban and odium of their displeasure, until another eighteen months has elapsed, and the eighth annual report issues from the parliamentary bill office. This report may possibly then announce that the recommendations here notified have been adopted. Assuredly this requires amendment. Again, in illustration of the inconvenience which must arise from the delay complained of, it is clear that the next report will embrace from the June of 1852 to the June of 1853, and not appear until about the same period—viz., the autumn of 1854, yet during that period the new Lunacy Bills will have come into operation, which will not therefore be officially reported upon until the tenth report, embracing the period from 1853 to 1854, makes its appearance. This, in the course of nature, will not happen until the autumn of 1855! This certainly is publishing "annual reports," as they are termed, with a heavy drag upon them. We observe that, by the 33rd section of the New Lunatics' Care and Treatment Bill, it is provided, in reference to the 88th section of the Act above cited, that these reports of the commissioners to the Lord Chancellor shall hereafter be "made on or before the month of March in every year, and shall be made up to the end of the preceding year;" but we do not perceive how this will facilitate or expedite their publication. We must, therefore, bear in mind that the Seventh Report of the Commissioners in Lunacy does not bring down the general statistics of insanity beyond the 1st of January, 1852; and the observations which they contain respecting the condition and management of certain asylums, must be received as applicable to them some *eighteen months ago*, and not as affording us any indication or criterion of *their present state.* It is only fair that this should be thoroughly understood; the grievance we recognise, but we do not see how it is to be remedied so long as these reports, prior to publication, are to be laid on the dormitory-table of the House of Commons, where measures not stimulated into active progression by the excitement of popular interests generally remain in a state of passive slumber during the greater part of the session.

We now proceed to analyse the report before us, viewing it in connexion with the report which was published by the commissioners five years ago—a comparison which will greatly enhance its interest. In the year 1847, referring our date always to the first of January, there were in the metropolitan district, which comprehends an area of some 15 miles round London, 45 houses licensed as private asylums, 9 of which were open for the reception of paupers. In the year 1852, the number of these licensed houses was 41, and only 5 appear to have been licensed for the reception of paupers. During the five years, therefore, the number of private lunatic asylums in the metropolitan districts has been reduced by 4; and, a circumstance we should not have anticipated, the number of lunatics confined in them has diminished by 141; the numbers male and female having in 1847 amounted to 2767, and in 1852, to 2326. We must not, however, infer that insanity has, during this quintennial period, been on the decrease, for, unhappily, when we look to the returns of the county asylums, hospitals, provincial licensed houses, and other places for the reception of lunatics, including Bethlehem, Haslar Hospital, and Thorncliffe Military Hospital, we find that between the year 1847 and 1852, there has been an increase of as many as 3580 lunatics; in 1847, there were returned 13,832; but in 1852, we find the number increased to 17,412. The number of persons found lunatic by inquisition does not present any corresponding increase, but appears—which is remarkable—to have remained almost stationary. Thus, in 1847, there were 235 persons found lunatic by inquisition, and in 1852 only 1 more, making 236. The tabular returns of criminal lunacy in the two reports exhibit an increase of 99; the numbers being in 1847, criminal lunatics, 337, and in 1852 increased to 436. The two summary tables, the one for 1847 and the other for 1852, may suggest other points for consideration; and we therefore here subjoin them.*

On the 1st of January, 1847, there were in England 20 county asylums; in Wales 1; and the number of lunatics therein confined amounted to 5486. At the commencement of the January of 1852, we find 25 county asylums open in England, 2 in Wales, and 4 borough asylums, giving an increased accommodation for 4731 lunatics, the total numbers confined being 10,217. It has long been the desire of the commissioners to insist upon every county providing one or more asylums for its own lunatic paupers. Hence, architects have been busy, and new county asylums are being erected in all directions.

In Lancashire, three county asylums—Lancaster, Rainhill, and Prestwich—are now open, which will, for many years, afford ample provision for the insane poor of this extensive and populous county. In Middlesex the additional asylum at Colney Hatch will also greatly relieve the county of its pauper lunatics; but from the rapidity with which it has filled, we apprehend that Hanwell and Colney Hatch will eventually be found inadequate for the reception of all the patients which will require admission. Under these circumstances the commissioners acted wisely in calling upon the Secretary of State—then Sir George Grey—to require the Court of Aldermen to provide an asylum for the pauper lunatics chargeable to the several parishes within the jurisdiction of the City of London. Under the new Lunatic Asylums Bill the justices of every county and borough not having a lunatic asylum are required to provide one; and we believe that ere long no private asylum will hold a licence for the reception of pauper lunatics. Our own opinion is, they are better separate. The system of organization required for the proper management of a pauper lunatic asylum differs in a great variety of details, from that which will be found the most eligible for the management of a private lunatic asylum; and although a feeling seems to exist with some persons that private asylums should be abolished, and accommodation provided in county asylums for the better class of patients, yet we have reason to know from the inquiries we have made, that the officers in charge of county asylums entertain a very strong conviction

* See next page.

*Summary Table for the 1st January, 1847.*

| — | Private. | | | Pauper. | | | Grand Total. | Found Lunatic by Inquisition. | | | Criminal. | | |
|---|---|---|---|---|---|---|---|---|---|---|---|---|---|
| | M. | F. | Total. | M. | F. | Total. | | M. | F. | Total. | M. | F. | Total. |
| County Asylums | 131 | 108 | 239 | 2431 | 2816 | 5247 | 5486 | 6 | 4 | 10 | 87 | 30 | 117 |
| Hospitals | 319 | 408 | 727 | 191 | 193 | 384 | 1111 | 13 | 5 | 18 | 4 | 1 | 5 |
| Metropolitan Houses | 548 | 555 | 1103 | 680 | 984 | 1664 | 2767 | 57 | 32 | 89 | 25 | 15 | 40 |
| Provincial Houses | 790 | 740 | 1530 | 1127 | 1205 | 2332 | 3862 | 77 | 42 | 119 | 51 | 13 | 64 |
| | 1788 | 1786 | 3574 | 4429 | 5223 | 9652 | 13,226 | 153 | 83 | 235 | 167 | 59 | 226 |
| Bethlehem Hospital | 112 | 138 | 250 | 104 | 36 | 140 | 390 | ... | ... | ... | 90 | 21 | 111 |
| Haslar Hospital (Naval) | 139 | ... | 139 | ... | ... | ... | 139 | ... | ... | ... | ... | ... | ... |
| Thorncliffe Hospital (Military) | 73 | 4 | 77 | ... | ... | ... | 77 | ... | ... | ... | ... | ... | ... |
| Totals | 2112 | 1953 | 4065 | 4533 | 5234 | 9767 | 13,832 | 153 | 83 | 235 | 257 | 80 | 337 |

*Summary Table for the 1st January, 1852.*

| — | Private. | | | Pauper. | | | Total Males. | Total. Females. | Total. Lunatics. | Found Lunatic by Inquisition. | | | Criminal. | | | Chargeable to Counties or Boroughs. | | |
|---|---|---|---|---|---|---|---|---|---|---|---|---|---|---|---|---|---|---|
| | M. | F. | Total. | M. | F. | Total. | | | | M. | F. | Total. | M. | F. | Total. | M. | F. | Total. |
| County Asylums | 131 | 120 | 251 | 4520 | 5446 | 9966 | 4651 | 5566 | 10,217 | 8 | 1 | 9 | 137 | 38 | 175 | 375 | 437 | 812 |
| Hospitals | 494 | 494 | 988 | 152 | 145 | 297 | 646 | 639 | 1285 | 11 | 5 | 16 | 8 | ... | 8 | 5 | 1 | 6 |
| Metropolitan licensed Houses | 568 | 570 | 1138 | 487 | 701 | 1188 | 1055 | 1271 | 2326 | 64 | 32 | 96 | 31 | 10 | 41 | 9 | 15 | 24 |
| Provincial licensed Houses | 727 | 721 | 1448 | 757 | 774 | 1531 | 1484 | 1495 | 2979 | 71 | 42 | 113 | 96 | 12 | 108 | 85 | 59 | 144 |
| | 1920 | 1905 | 3825 | 5916 | 7066 | 12,982 | 7836 | 8971 | 16,807 | 154 | 80 | 234 | 272 | 60 | 332 | 474 | 152 | 986 |
| Bethlehem Hospital | 193 | 190 | 383 | ... | ... | ... | 193 | 190 | 383 | 2 | ... | 2 | 85 | 19 | 104 | ... | ... | ... |
| Royal Naval Hospital, Haslar, Hants | 129 | ... | 129 | ... | ... | ... | 129 | ... | 129 | ... | ... | ... | ... | ... | ... | ... | ... | ... |
| Military Lunatic Hospital, Great Yarmouth. | 89 | 4 | 93 | ... | ... | ... | 89 | 4 | 93 | ... | ... | ... | ... | ... | ... | ... | ... | ... |
| Totals | 2331 | 2099 | 4430 | 5916 | 7066 | 12,982 | 8247 | 9165 | 17,412 | 156 | 80 | 236 | 357 | 79 | 436 | 474 | 152 | 986 |

that such a system would not work well, independent of which, persons of education and status in society will never consent to their relatives being domiciled under the roof of paupers, for however complete the separation may be as far as the architectural plan of the building is concerned, so long as the establishment is the same, the objection will prevail. The noble Earl who is the Chairman of the Board of the Commissioners in Lunacy, has, we regret to observe, pronounced in the House of Lords a very strong opinion upon this subject; and supposing that the Legislature in its wisdom did devise some such plan, the danger we apprehend would be that the old cottage system, which the philanthropic exertions of the noble Earl did so much to annihilate, will again be restored, inasmuch as persons who recoil at the idea of their relations being placed in a public asylum would prefer locating them elsewhere, and we have only to glance over the columns of the *Times* newspaper to see the number of "genteel private families," and "experienced physicians," advertising to take charge of lunatics, and the many *cottages ornées* which are open for their reception. In legislation this would be a retrograde step which, however, cannot be easily accomplished; indeed, we have no hesitation in pronouncing it wholly impracticable, for not living in a despotic country, the relations of the insane will very naturally insist on their feelings being consulted in providing for such afflicted persons. Whatever prejudices may be entertained against private asylums, their existence we believe to be absolutely necessary; and all the Legislature can do is to secure for them such an efficient system of visitation and supervision as shall ensure their being conducted on the best and the most humane principles. The argument urged against them, that the proprietors have a pecuniary interest in these establishments, admits of the very contrary application; the abuses which are found to exist at certain times in great public institutions may almost invariably be traced to the fact of an irresponsible management; and if the persons guilty of negligence, and a thousand irregularities, had only a *bonâ fide* interest in such concerns, they would not, for their own sakes, allow the shadow of a reproach to fall upon them. The best guarantee that the public can have that an establishment shall be well conducted may be looked for in the fact that the proprietors have a pecuniary interest in them; for it is universally acknowledged that the path of rectitude leads to fortune, and that of roguery to inevitable ruin. The very converse of the proposition, therefore, enunciated against them, holds good. Where a man has an interest in an establishment—where his responsibility is declared—he is far more likely to exert himself conscientiously and to the best of his ability, than when he has only a stipendiary interest in an establishment and shares a divided responsibility. The Commissioners in Lunacy do not, it is to be hoped, entertain the opinion of the noble Earl at the head of their Board, otherwise painful, indeed, must be the discharge of their duties in supervising a system which they at the same time desire abolished. This, however, we do not believe. To return, however, to the Report before us. Having notified the changes which have taken place in the metropolitan and provincial licensed houses—the new county asylums which have been opened, and those which are far advanced towards completion, and which will have the effect of closing numerous asylums for the reception of pauper lunatics—the commissioners state that applications are occasionally made to them, on the part of committees of visitors engaged in organizing lunatic asylums, for their advice respecting the organization of the staff of officers; and they, therefore, some time ago, circulated a paper of suggestions, of which the following is a copy.

### Commissioners' Suggestions for the Staff of a County Lunatic Asylum.

### *Resident Medical Superintendent.*

This officer should be duly qualified both as a surgeon and apothecary (whether possessing a degree or not).

He should have *paramount* authority in the asylum, and be precluded from private practice, and should devote his whole time and energies to the duties of his office.

He should be a person of high character and experience, and be liberally remunerated.

With regard to salary, the commissioners find that in the various county asylums the salaries vary from one hundred and fifty to five hundred pounds per annum, to which is to be added, where (as is commonly the case, and is always most desirable) he resides on the premises, furnished apartments, with coals, candles, and generally also board, or an equivalent in money for the same.

The commissioners consider it the preferable arrangement that there should not be any visiting physician or other medical visitor with a salary, but that in lieu thereof the resident medical superintendent should have the power to call in medical or surgical advice, on extraordinary occasions, at the expense of the asylum. If there are honorary physicians or surgeons attached to the institution, their services would be gratuitous, unless when they were so specially called in.

In the event of the asylum becoming full, or nearly full, it may be advisable to appoint an assistant medical officer, with board and lodging, at a moderate salary, say from fifty to eighty pounds per annum. Such an arrangement has been adopted in many of the large asylums, *e. g.*, Lancaster, Kent, Stafford, &c.

### *Resident Clerk and Steward.*

The commissioners are disposed to think that these offices may be conveniently joined, and that, at least until the number of patients be considerable, this officer may (in addition to his ordinary duties) examine, from time to time, or daily, the condition of the bedding and clothing, and generally overlook the male side of the establishment. He should have suitable board and lodging in the asylum. The salary of this officer varies in different asylums from fifty to one hundred pounds, according to the position assigned to the person holding the office, and the amount of duty imposed upon him.

### *Matron and Housekeeper.*

She should be a single woman (or widow) of the middle class of life, of active business habits, and capable of personally directing all the details of a large household, such as the kitchen, laundry, cutting out and making up dresses, clothing, &c., and directing the female patients in their needle work, &c., in addition to the superintendence of the female wards, nurses, and female servants.

A person brought up with the habits of a lady appears to be in general not desirable.

Her office would in a great degree combine the duties of a house-keeper and those of head nurse, and her salary should be not less than double the amount of wages of any other nurse, together with apartments and board. In the generality of existing asylums it varies from forty to one hundred pounds a year.

### *Chaplain.*

The salary of this gentleman, who will, it is presumed, be a non-resident officer, may be generally described as equivalent to that of a curacy. In the different asylums it usually varies between fifty and one hundred pounds a year. In two or three asylums, however, it exceeds this amount. In the Middlesex Asylum at Hanwell, where there are nearly one thousand patients, it is two hundred pounds; and in that of Lancaster, where the patients are nearly eight hundred, it is three hundred pounds a year.

The particular amount ought, in some degree, to be regulated by a consideration of the quantum of labour imposed, and the amount of time required for the due performance of the duties.

The practice has hitherto, we believe, been that the chaplain should not reside in the asylum, and a contrary practice would seem in general to be open to grave objections.

*Treasurer.*

Some local banker would, doubtless, perform the office of Treasurer without salary.

*Clerk to the Visitors.*

A fixed payment for each official visit to the asylum with the Visiting Justices, or a yearly salary, not exceeding in either case a limited and very moderate sum, appears to the commissioners to be the best mode of remunerating the labour incident to this office. Possibly, however, with a view both to convenience and economy, a satisfactory arrangement might be made for having the duties performed by the clerk of the asylum.

*Male and Female Attendants.*

In addition to the qualities obviously requisite in servants of this class (such as good temper, patience, sobriety, honesty, activity, and general intelligence), it is desirable that each attendant should be able to read and write, and should be qualified to train and direct the patients in their various in-door and out-door occupations.

The commissioners think that the wages of the attendants should be liberal, and should be subject to periodical increase in cases of length of service and good conduct.

Before making any comment upon these suggestions, we shall lay before our readers the subjoined Table, which we have been at the pains to construct from the Report before us. It exhibits the amount of salaries which the officers referred to now enjoy at the different county asylums specified. The asterisk signifies non-resident. The first column states the number of patients in the asylum on the 1st January, 1853, with the view of giving some idea of the amount of duty required.

### TABLE OF SALARIES GIVEN TO THE SUPERIOR OFFICERS IN COUNTY LUNATIC ASYLUMS.

| | Number of Patients. | The Medical Staff. | Superintendent. | Chaplain. | Matron. | Assistant Matron. | House-keeper. | Clerk to Visitors. | Clerk and Steward. | Steward. |
|---|---|---|---|---|---|---|---|---|---|---|
| **Bedford** . . . . | 267 | . . Resident Medical Officer, £100, board | £150* | £80* | £30, board | £18, board | . . . . | £100* | | |
| **Cheshire** . . . . | 225 | . . Physician and General Superintendent, £300.* Assistant Medical Officer, £100, board, &c. | . . . . | £70* | . . £40, board, &c. | . . . . | . . . . | £80* | | |
| Cornwall Asylum . | 231 | . . Medical Superintendent, £315 . . Visiting Physician, £100* | £200 | £50* | . . Not determined. | . . . . | . . . . | £40* | . . . . | £50 |
| Denbigh or North Wales Asylum . | 156 | . . Medical Superintendent, £150 . . | . . . . | £50* | £50 | . . . . | . . . . | . . . | £100 | |
| Derbyshire Asylum . | 77 | . . Medical Superintendent, £400, board, residence, washing, fuel, and light. | . . . . | £60* | . . Included in Superintendent's salary and allowances. | . . . . | . . . . | . . . | . . £140, residence, fuel, light. | |
| Devon Asylum . . | 429 | . . Medical Superintendent, £500, residence and washing. Medical Assistant, £100, board, residence, and washing. | . . . . | £150* | . . . . | . . . . | . . £40, board, residence. | £60* | £105 | |
| Dorset Asylum . . | 158 | . . Medical Superintendent, £200, board, and residence. | . . . . | £50* | . . . . | . . . . | . . and cook, £30, board, residence. | . . . | . . £30,* clerk only. | |
| Gloucester Asylum . | 311 | . . Physician and Superintendent, £300, suite of apartments, fuel, and lighting. Medical Assistant, £50, board and washing. | . . . . | £60* | . . £100, wife of Physician. | . . £25, board and washing. | . . . . | . . . | £140* | |
| Kent Asylum . . . | 498 | . . Visiting Physician, £100* . . . . Resident Medical Superintendent, £400, mats and matting, coals, candles, oil, soap, milk, garden produce, washing for self and family. | . . . . | £150* | . . . £50, apartments furnished, with board, washing, and attendance. | . . . . | . . . . | . . . | . . £170, residence, coals, candles, oil, soap, milk, produce of garden for self and family, and of farm for keep of pony. | |

| | | | | | | | | | | |
|---|---|---|---|---|---|---|---|---|---|---|
| Lancaster Asylum . | 679 | . . Physician, £150* . . . . . . . . House Surgeon, £120, board and lodging. | . . £400, house furnished (except plate, glass, and china), fruit &vegetables. | £300* | . . £75, board and lodging. | . . £22, board and lodging. | . . . . | . . . | . . . . . | . . £80* unfurnished house. |
| Rainhill Asylum, near Liverpool. | 296 | . . Medical Superintendent, £350, house, coals, gas, soap, candles, milk, vegetables. House Surgeon, £50, board and lodging. | . . . . | . . £200, house, and garden. | . . £75, board and lodging, | . . £25, board and lodging. | . . . . | . . . | . . £75, board and lodging. | |
| LANCASHIRE— Prestwich Asylum, near Manchester. (*a*) | 302 | . . Resident Medical Officer and Superintendent, £350, unfurnished house, coals, gas. House Surgeon, £60, furnished apartments and board. | . . . . | . . £200,* unfurnished house, coals, gas. | . . £50, furnished apartments and board. | . . . . | . . . . | £40* | . . . . | . . £70 apartments and board. |
| Leicestershire and Rutland Asylum. | 238 | . . Medical Superintendent, £300, board, lodging, washing, all necessaries, except wine. | . . . . | £40* | . . £52 12s. board, lodgiug, washing. | . . . . | . . . . | . . . | . . Clerk & legal adviser, £30. | |
| Lincolnshire Asylum | Not yet open. | . . Resident Medical Superintendent, £500. | | | | | | | | |
| MIDDLESEX— Colney Hatch. (*b*) | 1005 | . . Medical Officer (male side), £200, house, coals, candles, milk, vegetables, and £150 a-year in lieu of board. Medical Officer (female side), ditto. Dispenser, £50, board, lodging, and washing. | . . . . | . . £200,* an unfurnished house. | . . £150, furnished house, coals, candles, milk, vegetables, and £80 a-year instead of board. | . . £30, board, lodging, & washing, | . . £25, board, lodging, & washing. | £100* | . . Clerk, £200; Assistant Clerk, £70; ditto, £25; board, lodging, & washing. | . . Steward's Clerk, £70. |
| Hanwell Asylum. (*c*) | 961 | . . (*d*) Resident Medical Officer, £200, board, lodging, and washing. Ditto (female side), £200, board, lodging, and washing. Dispenser, £70, ditto. | . . . . | £250* | . . £200, board, lodging, & washing. | . . £60, board, lodging, & washing. | . . £30, lodging, & washing. | £100* | | |

(*a*) The Treasurer and Clerk to this Asylum receives a salary of £120 per annum.
(*b*) The Assistant in Stores to this Asylum receives a salary of £25, with board, lodging, and washing.
(*c*) The Schoolmaster in this Asylum receives a salary of £90.
(*d*) The Office of Visiting Physician, whose salary was £315 per annum, has been abolished.

TABLE OF SALARIES—*continued.*

| | Number of Patients. | The Medical Staff. | Superintendent. | Chaplain. | Matron. | Assistant Matron. | House-keeper. | Clerk to Visitors. | Clerk and Steward. | Steward. |
|---|---|---|---|---|---|---|---|---|---|---|
| Monmouth, Hereford, Brecon, Radnor, joint Counties Asylum, Abergavenny. | 32 | . . Physician and Superintendent, £250, with board and apartments. | . . . . | £100* | . . £40, board, apartments. | . . . . | . . . . | . . . | . . £80, board and apartments. | |
| Norfolk Asylum . . | 275 | . . Visiting Surgeon, £84* . . . . . . Resident Surgeon, £100, board and lodging. | . . £150, board and lodging. | £80* | . . £50, board and lodging, | . . £16, board and lodging. | | | | |
| Nottingham Asylum | 236 | . . Physician, £100* . . . . . . . . Medical Superintendent, £200. | . . . . | £70* | £80 | . . . . | . . . . | . . . | £70 | |
| Oxfordshire & Berks Asylum. | 347 | . . Medical Superintendent, £250, furnished apartments, board, &c. Clinical Clerk, £70, ditto. | . . . . | . . £140, unfurnished house. | . . . . | . . . . | . . £30, furnished apartments, board. | . . . | . . £70, furnished apartments, board. | |
| Salop and Montgomery Asylum. | 216 | . . Medical Superintendent, £300, self and wife maintenance. | . . . . | £40* | See Superintendent. | . . . . | . . . . | . . . | . . Steward, £42;* Clerk, £50.* | |
| Somerset Asylum . | 340 | . . Medical Superintendent, £500, house furnished, washing, coals, candles, vegetables. Surgeon, £70, with furnished apartments, washing, firing. | . . . . | £126 | . . £45, apartments, and room and a servant. | . . . . | . . . . | . . . | . . Clerk & Steward, & Clerk to the Visitors£70; apartments, washing, firing. | |
| Stafford Asylum . . | 349 | . . Visiting Physician,* £200,* fees in addition upon all private patients. Medical Officer, £400, board & lodging. | . . . . | £80* Clerk, £25. Organist, £10. | . . £100, board and lodging. | . . . . | . . £30, board & lodging. | | | |
| Suffolk Asylum . . | 241 | Superintendent and Matron, £450 . . | . . . . | £60.* | . . See Superintendent. | . . . . | . . . . | . . . | . . . . | £43. |

| | | | | | | | | | | |
|---|---|---|---|---|---|---|---|---|---|---|
| Surrey Asylum . . | 853 | . . Visiting Physician, £210 . . . . Two Resident Medical Officers, £350 each—£700; furnished apartments and coals. | . . . . | £100* | . . £120, furnished apartments and board. | . . £60, furnished apartment, and board. | . . . . | . . . | . . £330, furnished apartments and coals. | . . Steward's Clerk, £70. |
| Warwick Asylum . | Not yet open. | . . Resident Medical Superintendent, £300, furnished apartments, fire, light, and washing. | . . . . | £60* | £60 | . . . . | . . . . | . . None fixed. | . . £100, furnished house, fire, light, and washing. | |
| Wilts Asylum . . . | 165 | . . Medical Superintendent, £400, and £100 in lieu of board; attendance, washing, vegetables from the garden. | . . . . | £100* | . . £50, furnished apartments, board & washing. | . . . . | . . . . | . . . | . . £70, furnished apartments, light, & washing. | |
| Worcester Asylum . | Not yet open. | . . Medical Superintendent, £350, furnished house, coals, washing for family. | . . . . | . . . . | . . £60, board, lodging, washing. | . . . . | . . . . | . . . | . . £100, furnished house, gas, coals, & vegetables. | |
| YORKSHIRE— North-East Riding Asylum | 271 | . . Medical Superintendent, £320, and £100 with farm and garden produce, in lieu of rations. | . . . . | £80* | . . . . | . . . . | . . . . | £21* | . . £100, with rations. | |
| West-Riding Asylum | 611 | . . Two Visiting Physicians at £105 each—£210.* Medical Superintendent and Treasurer, £400, with board and lodging. Apothecary, £70, board and lodging. | . . . . | £250* | . . £150, board and lodging. | . . £30, with board & lodging. | . . £30, with board & lodging. | . . . | . . Clerk, £65, board and lodging. | |
| Birmingham Borough Asylum. | 263 | . . Medical Superintendent, £350, house, firing, and gas. | . . . . | £100* | . . £60, board, lodging, washing | . . . . | . . . . | . . . | . . £125, board, lodging, washing. | |
| Bristol Asylum . . | 73 | . . Visiting Physician, £75.* . . . . House Surgeon, £50. | . . Superintendent and Clerk, £50. | £60* | . . £25, board and lodging. | . . . . | . . . . | £50 | | |
| Haverfordwest Asylum. | 33 | Medical Officer, £20 . . . . . . . | . . . . | . . . . | . . £28 12s, maintenance, fire & candle. | . . . . | . . . . | £5 | | |
| Hull Borough Asylum | 79 | . . Medical Officer, £230, house, fuel, and vegetables. | . . . . | £50 | . . £25, board and lodging. | . . . . | . . . . | . . . | £40 | |

We have always considered that the organization of the medical staff connected with our principal lunatic asylums is very defective, particularly when compared with the great continental asylums, the *Salpétrière*, the *Charenton*, and the *Bicêtre*. Upon referring to the table before us, it will be seen that the Kent Asylums, containing 498 patients; the Nottingham Asylum, 236; the Stafford Asylum, 349; the Surrey, 853; and the Bristol, 73 only, has each an appointed visiting physician; and that the Norfolk Asylum, having 275 patients, has a visiting surgeon; and the Yorkshire West Riding Asylum, with 611 patients, has *two* visiting physicians; and yet our two metropolitan asylums—Hanwell, with its 961, and Colney Hatch, with 1005 patients in it—have neither of them a consulting physician officially connected with them. The commissioners state that they consider it a preferable arrangement that there should not be any visiting physician, or other medical visitor, with a salary, but that in lieu thereof the resident medical officer should have the power to call in additional medical or surgical advice on extraordinary occasions, at the expense of the asylum. They, however, concede that honorary physicians may be attached to such institutions—but in that case, unless specially called in, their services should be gratuitous. This is a question which we conceive ought not to be dealt with upon mere *financial principles;* if it be desirable that visiting physicians should be attached to the medical staff of our county asylums, the expediency of such appointments should be recognised. Unfortunately, as Sir George Ballingall has so well shown in his little brochure upon the construction and arrangement of Medical Hospitals, so large a sum of money is expended for the most part in unnecessary *architectural* display, that the resources of such institutions are often found to be crippled when it becomes necessary to draw upon them for the necessary purposes of the charity. We observe by the Table before us that some of the county asylums, erected in the style of palaces, at an enormous expenditure, have so impoverished their exchequer, that, while vestry committees are complaining, and rate-payers groaning, they can only afford to pay the resident medical officer—the most important functionary of the institution—a pittance of 200*l*. a year. We do not, therefore, we repeat, deal with it as a question of finance; all we contend for is, that our metropolitan and county lunatic asylums should be put on the same footing, and that their medical staff ought to be organized on the same principles as we find at other public hospitals. If Guy's or St. George's Hospital has each its staff of consulting physicians and surgeons, why should not Hanwell and Colney Hatch Asylums, which have conjointly charge of two thousand patients, or more, be provided with as liberal a medical staff? Or, looking to the provinces, if the Bedford General Infirmary, or the Cheshire General Infirmary, has each its consulting physicians, why should not the lunatic asylums of those counties have medical officers of analogous *status* attached to them? Why should these asylums, as far as the medical staff is concerned, be organized on narrower principles than even the medical dispensaries in every city and borough town in the kingdom? Is the treatment of disorders of the mind or brain of less importance to the community than that of diseases known to be infinitely more curable and less distressing? If the Liverpool dispensaries have some half-dozen consulting physicians attached to them, why should not the County Asylum of Rainhill, only a few miles distant, organized for the reception of more than 500 patients, not have *one* connected with its staff? The public hospitals of this country, whether instituted for the cure of diseases of the body or the mind, ought to have the most eminent medical practitioners in the profession appointed to them. The arrangement, to secure their services, should be permanent and not fleeting, temporary or optional. In general private practice, either the rank of the patient, or the expressed wishes of the family generally suggest, in doubtful or difficult cases, the propriety and expediency of calling in additional advice. Why should not the poor inmates of a hospital, whose indigent relations cannot originate such a suggestion, be provided with the same advantage of having the best professional advice that can be commanded at hand to assist them? Does not humanity suggest that the most experienced and skilful medical practitioners in the kingdom should be appointed to these institutions? Assuredly it does; and the high reputation of the physicians and surgeons connected with our public hospitals sheds a lustre on these noble charities, which greatly tends to promote their prosperity, and realize the objects which the munificent founders and donors to them originally contemplated. Were there no such official appointments, no consulting physicians permanently attached to these hospitals, many of them would lose the *prestige* which they now enjoy, and with it the confidence of the public. When the commissioners suggest that, instead of there being any visiting physician officially attached to lunatic asylums, and recommend that medical superintendents should, in extraordinary cases only, call in additional advice, they forget that their recommendation of the abolition of the office seems itself to imply that such consultations are, in their opinion, very seldom, if ever, required. We, indeed, believe that, if left optional with medical superintendents, the opinion of a consulting physician will seldom or never be sought, *as it ought to be in the ordinary routine of professional duty*. The appointment has been abolished at Hanwell Asylum; and what will be the result? We are not prophets, nor are we gifted with the mesmeric faculty of prevision, but we will hazard a conjecture that the late consulting physician to this asylum will not, upon the freetrade principle, be called in to give the benefit of his opinion and advice in as many cases during the next five years as he was in the habit of seeing in one week—we should rather say in a single day—during the period he held his official appointment. We believe that the medical superintendents and medical officers connected with public and private lunatic asylums are, for the most part, very well informed and practically competent men; but, without the least disparagement to any of them, we still think the addition of visiting or consulting physicians to the ordinary medical staff very desirable. To the medical superintendents themselves, individually and collectively, it would be a very great advantage; in cases which are not very extraordinary, but still perplexing, they would, without going out of their way, have additional advice at hand; they would find themselves thereby relieved from much responsibility, and supposing we regard the consulting physician as holding a higher professional *status* than his junior officers, the recognition of the office would, among them, be an incentive to further zeal and assiduity, and an encouragement to honourable emulation. In one speciality it is well known that the best and the highest class of psychological physicians do not, for reasons that must be obvious to our readers, offer themselves for the post of resident medical officers to our county lunatic asylums; many of them may have gone through this probation, and there acquired their knowledge, their experience, and their fame; after all which, we may fairly ask, is the highest rank they can arrive at in this department of the profession to be cut down to the level of a medical superintendentship, a compulsory residence in an asylum, upon an income of two or three hundred a-year?

When, indeed, we look over the table before us, it is sad to observe how small is the *unincreasing* salary of many, doubtless, able, well-informed, and accomplished medical men, holding, as resident superintendents, an office which entails upon them much personal confinement—amounting almost to a total deprivation of liberty—unceasing toil and ever-fluctuating anxiety. When years have thus elapsed—and the prime of life is almost gone—what have they gained? no pecuniary fortune certainly—and as little honour if they cannot attain the recognised *status* of being consulting physicians in their own speciality. Are not the observations of the commissioners, in reference to the inexpediency of appointing visiting physicians to the county lunatic asylums calculated to inflict an injury upon the profession? The office of visiting physician to county asylums being declared by them unnecessary, the most experienced physicians in lunacy would thereby be precluded from connecting themselves in that capacity with these institutions, and therefore cannot hold a position which their medical brethren—eminent in other paths of the profession—enjoy, in being officially connected with other hospitals, where they have the opportunity of exercising their professional skill with honour to themselves and with advantage to the public. With the other suggestions of the commissioners we cordially agree, and particularly with the observation that "when a medical man is elected to any new asylum he should enter upon his duties some months before the building is open for the reception of patients." They truly observe that "the amount of additional salary so incurred will be far more than saved if, during the intermediate period, the committee have the benefit of his active services, and advice in the laying out of the yards and grounds, in the fitting up, and furnishing of the asylum, and in selecting the subordinate officers and attendants, and regulating their respective departments and duties."

The commissioners next direct the attention of the Lord Chancellor to the subject of the boroughs—in number upwards of fifty—including the City of London, which have not yet made legal or adequate provision for their pauper lunatics; but we have no doubt that when the new Lunatic Asylums Bill has come fairly into operation, the different boroughs and counties which are now without asylums will be obliged to provide them, however unwilling some of the local authorities may be to incur such expense. Indeed, the report before us furnishes us with conclusive evidence that the Commissioners in Lunacy frequently find their recommendations disregarded, and their wishes thwarted by the justices of certain boroughs and counties. It is an old grievance, and one which may be easily understood. Under the present system, two authoritative powers co-exist in the provinces; the justices at quarter sessions grant the licences of lunatic asylums, the management of which is under their immediate jurisdiction. They visit the licensed house every three months, or perhaps much oftener, accompanied with their medical officer, and make their entries in the visitors' book and the patients' book. The administration of the law being vested in the commissioners, they also have the powers of supervision; and in the meantime, twice a year, or oftener, they make their visit; and without reference to the magistrates they inscribe their own remarks and recommendations as entries in the same books: what follows? The visiting justices not only frequently disregard the suggestions of the commissioners, but in some instances they set their authority at nought. Thus, in the report before us, the commissioners give a detailed statement of their contumacious treatment by the justices of the Hull Borough. In vain, since the opening of this asylum, did they repeat their visitations, and remonstrate against defective accommodation and general mismanagement; the attention of the Board in New-street, Spring-gardens, was in vain called to the pertinacious obstinacy of these justices, until, wearied with official correspondence, and "having no reasonable expectation," they state, "that any steps would be taken by the committee of visiting justices to the remedy of the manifest defects," upon which they had animadverted, they inform us that they had recourse to the *dernier ressort*—an appeal to the Secretary of State —and with what success? The Secretary of State for the Home Department transmitted, we are told, the document which the commissioners had drawn up, reciting the causes of complaint, down to the visiting justices for their information, and "they in reply, after denying the necessity for many of the changes and improvements recommended by the commissioners, stated that several of their recommendations had been carried out." Here, for awhile, the matter rested, after which the report informs us that "the asylum was again, in August, 1851, visited and animadverted upon by two of the commissioners, who reported that its construction and general arrangements were *the same* as noticed in previous entries." The visiting justices, be it observed, had already treated their previous recommendations with the most perfect indifference—and thereupon it seems the commissioners again made a variety of suggestions for the improvement of the institution—and again *without effect*, inasmuch as they state that "At the last visit made to the asylum on the 4th March last (1852), the visiting commissioners reported that although some few additions had been made, the asylum was substantially in the same state as before. Upon the perusal of this report, the Board addressed a letter to the visiting justices on the 28th March, pressing for a general reviewal of the whole arrangements and economy of the asylum, and offering every assistance on the part of the commissioners—*and to this communication the Board have as yet received no reply beyond the acknowledgment of its receipt.*" The commissioners add, addressing (as their report does) the Lord Chancellor, "Your Lordship is aware that as the Hull Lunatic Asylum is a borough asylum (established under the provisions of the Act 8 and 9 Vict. c. 126), we have no means of compelling the justices of the borough to adopt our recommendations, however beneficial they might prove to the patients, otherwise than by calling in the assistance of the Secretary of State." But the assistance of the Secretary was confessedly called in by the commissioners—their statement was transmitted to the justices, and the justices in their turn remonstrated, and after all still set the authority and recommendations of the Commissioners in Lunacy at defiance.

But this is not the only part of the country in which this species of "flat rebellion" appears to have been committed. We find by this same report that the justices of the city of Norwich have been equally, if not more refractory, although the extracts which the commissioners have given of their two last entries clearly enough establish the existence of defects and abuses at the asylum at Norwich which ought not to have been tolerated; yet, after all the visitations and official remonstrances of the commissioners, the justices appear to have made no manner of concession, and the report of the commissioners before us winds up with this *brutum fulmen*:—"Should the justices of the city of Norwich persist in neglecting to make a fit provision for their lunatic paupers, conformably to the directions of the Act 8 & 9 Vict. c. 126" (sect. viii. of the new Lunatic Asylums Bill), "it will be our duty to bring the subject of borough asylums again under the notice of the Secretary of State."

Our object in calling attention to these cases is to show, that although, speaking generally, we believe the justices of her Majesty's Commission throughout the country, willingly co-operate with the Commissioners in Lunacy, they in some instances act otherwise, and even appear to be in direct antagonism with them. This is to be regretted, and evinces the inexpediency of a twofold authority which does not act in unison—and the obvious consequence is, that the managing officers of such asylums pay more deference to the visiting justices than to the commissioners—in whose hands, notwithstanding their supervision, little immediate power is placed. The power which the visiting justices possess consists in their being able to withhold at quarter sessions any particular licence, in the granting of which not only have the commissioners no influence, but their advice *not* to grant a licence to certain houses has, in some cases, been disregarded, and the licence granted in the very teeth of their disapproval.* Who can read, without perceiving the force of these remarks, the following extract from the Report before us?

"The licensed house called Castleton Lodge, near Leeds, has of late years been frequently the subject of our animadversion, although no particular case of abuse has occurred which has justified us in condemning it altogether as unfit for a licence.

"In the opinion of the visiting commissioners, the establishment is gloomy and defective in cleanliness. It is unfit for the accommodation of as many patients as are permitted by the licence to be received therein; patients are allowed to be in bed, without having any bodily ailment, and the art of judiciously managing the patients seems not to be understood, or at least very little practised by the proprietors of the house.

"*Unfortunately the local visitors do not appear to concur in the views which we have here expressed of the short-comings of Castleton Lodge as a place for the treatment of insanity, and we regret to be obliged to add that, in the abseene of their cordial co-operation, our own efforts towards*

* See the last Annual Report of the Commissioners, (the *sixth*, page 12,) where it is stated that a letter was addressed by the Board of Commissioners to the Chairman of one of the Quarter Sessions, requesting the justices not to grant or renew a licence to a certain asylum, upon which occasion they state "no reply was received from the justices to their communication, and the *licence was renewed as usual.*"

*correcting its various defects and enforcing a generally improved system of management have been attended hitherto with but indifferent success.*"—p. 28.

It appears to us, that if the commissioners are to have a supervision and jurisdiction over the management of provincial licensed houses they ought to have a voice in the renewal or non-renewal of their licences, which, at all events, ought not to be granted in direct opposition to their sanction and authority. The appeal to the Secretary of State does not, it is clear, from the evidence in the Report before us—readily assist them, but only entails upon them a long official correspondence, which does not lead to any satisfactory result. The Lunatic Asylums Bill, however, has now come into operation, and it remains to be seen how far the justices will consult the opinion or defer to the judgment of the commissioners in erecting new lunatic asylums, and in providing adequate accommodation for their pauper lunatics.

Within the circuit of the metropolis are the registered hospitals of St. Luke's, Guy's, and Bethlehem. The commissioners state that many of the defects which they had occasion to notice in previous reports in the management of St. Luke's Hospital, have been remedied; but they still complain that the hospital continues to be placed under the medical care of only *one* resident surgeon and apothecary, and that the two physicians and surgeon, who are also on the medical staff, although they have considerable power, do not reside there. They emphatically recommend that here, as at Bethlehem, the medical staff should comprehend the services of at least *two* medical officers, one of whom should have paramount authority. They reiterate their opinion, which we apprehend cannot in principle be contravened, that in all the various lunatic hospitals, and public asylums of the country, the paramount authority should be invariably vested in some *resident medical officer* rather than any non-resident physician or surgeon, whose means of observing the wants and condition of the patients must obviously be very imperfect. So thoroughly do we concur in this opinion, that we are often surprised to find the commissioners sanction the residence in asylums of non-medical superintendents, and, above all, that women should be given licences for keeping asylums. By the present Report it appears, that during the year to which it refers, five new licences have been granted in the metropolitan district, three of which have been given to women, which certainly seems very contradictory—or, at all events, not in accordance with the opinion here so strongly expressed. The commissioners express themselves well satisfied with the improvements which have since their last annual report been introduced into the ward appropriated to insane patients at Guy's Hospital; but announce that the project of establishing a lunatic asylum for the reception of the patients, in a more suitable site in the county of Surrey, is under the consideration of the governors of the hospital. We have next in this Report a detailed account of the different steps taken by the commissioners in communicating with the Secretary of State and the governors of Bethlehem Hospital respecting the state of Bethlehem; and we have no hesitation in saying they appear throughout to have acted with the greatest consideration and courtesy towards Sir Peter Laurie, as the President of the Board of Governors, and the other governors of the hospital. As, however, the commissioners have succeeded in getting the hospital placed under their immediate jurisdiction, which it now is by the 35th section of the Lunatics' Care and Treatment Bill, and the controversy therefore is at an end, no advantage can arise from recapitulating details which cease to possess any further interest. We believe the resident physician of Bethlehem Hospital hails with satisfaction the circumstance of the institution being placed under the supervision of the commissioners, which must, viewed in a proper light, be a great protection to every lunatic asylum.

From the review which the commissioners have taken of the entries made in the books of the provincial and metropolitan asylums, during the twelve months included in this Report, it does not appear that any very serious defects or mismanagement were found to exist in any of them. This is a very gratifying circumstance, and speaks volumes in favour of the resident medical officers and proprietors of these establishments. The censorious animadversions which are passed upon some few houses, we pass over, thinking it probable, as we have already premised, that during the interval which has elapsed between the date of these entries and the publication of this Report, the proprietors of these asylums may have removed the causes of such complaints. While in all public and private lunatic asylums great improvements have been effected, the commissioners state with regret that the law respecting *single* patients in unlicensed houses continues most unsatisfactory, and they further attest the impossibility of more than a small proportion of them being visited by the private committee, the duties of which must, it is evident, be as multifarious as they are onerous. We may add, that we have reason to know that there is a very great difficulty in bringing the provisions, perhaps of any act of parliament, to bear upon individual and private cases. Look, we repeat, at the advertisements in the *Times* newspaper—it is not reasonable to suppose that the "experienced medical practitioners," widows, and private families, daily advertising to take charge of invalids afflicted with nervous and mental complaints (*anglice*, lunatics) will voluntarily place themselves under the jurisdiction of the Commissioners in Lunacy. They take individual patients and say nothing about it—nay, the relations of some insane persons, under the old notion that insanity inflicts a ban upon the family, stipulate against such reports being made. They are unwilling the affliction should be made known, and bargain for secresy being an element of their agreement. Here then is an evil which the law confessedly cannot reach; for it is certain that single patients are more liable to be maltreated than several patients domiciled under one roof. Numbers give individual protection, which is further guaranteed by the supervision of the commissioners; but this very grievance, the evil of taking single patients, would, we submit, be greatly increased if private asylums were abolished, and the attempt made to constrain persons to send their relations to public lunatic asylums. Rather than submit to so obnoxious a law, which, as we have explained, would, in many cases, be very revolting to their feelings, they would have recourse to any alternative, and the cottage treatment would be revived with all its manifold abuses. We believe one of the soundest principles of legislation is, not to enact any law the operation of which may suggest its own violation; and we believe, by giving the public confidence in the management of private asylums, the importance of early treatment in cases of insanity will be recognised, and these single patients drawn out of their hiding places. The commissioners, during the period to which the Report refers, have not found it necessary to institute more than three prosecutions against persons attempting to keep lunatics in unlicensed houses—and in each case they obtained a verdict. Their Report concludes with observations upon the expediency of establishing a separate asylum for the confinement of those patients usually described under the objectionable appellation of "criminal lunatics." It appears that Lord Shaftesbury, as Chairman of their Board, having promised to bring the matter under the consideration of the House of Lords, they have, with the view of obtaining as many particulars as possible, addressed circulars, containing various inquiries, to the visitors of county lunatic asylums, and the superintendents and proprietors of hospitals and licensed houses. They requested, first, a return of all patients confined under the Royal authority or Secretary of State's warrants, or confined by order of justices, as persons apprehended under circumstances denoting a derangement of mind and a purpose of committing an indictable offence; and, secondly, their opinion on the subject of criminal lunatics generally being allowed to associate with the ordinary inmates of asylums. They then enumerate the main objections to the association of the two classes of patients, which may be briefly stated as follows:—

"1. That such association is unjust, and that it gives pain and offence to ordinary patients (who are generally very sensitive to any supposed degradation), and also to their friends.

"2. That its moral effect is bad, the language and habits of criminal patients being generally offensive and their propensities almost invariably bad. That in cases of simulated insanity (which seem to be not infrequent) the patient is generally of the worst character, and that even where the patient is actually insane the insanity has been often caused by vicious habits. That patients of this class frequently attempt to escape, and cause insubordination and dissatisfaction amongst the other patients.

"3. That a necessity for stricter custody exists for one class than for the other, and that this interferes with proper discipline, classification, and general treatment, and strengthens the common delusion that an asylum is a prison.

"4. That criminal patients concentrate attention on themselves, and deprive the other patients of their due share of care from the attendants.

"5. That the effect on criminal patients themselves is bad; that they are taunted by the other patients, and are irritated on seeing such other patients discharged."—p. 33.

We must consider that the whole of this very important subject is at present *sub judice.* It will, doubtless, next session, be brought prominently before the Houses of Parliament; in the meantime the commissioners have in this report furnished us with a summary statement in a tabular form of the numbers, classes, and sexes of criminal lunatics confined in asylums, lunatic hospitals (including Bethlehem), and licensed houses in the month of March, 1852. By this return it will be seen that the number of patients falling within the designation of "criminal lunatics" (which the commissioners allow is, in many cases, a very inappropriate designation) amount to 439, of which, 360 were males, and 79 females. The number charged with felonies against life was 138; and against property 188. We conclude our observations by subjoining the Table.

### CRIMINAL LUNATICS.

*Abstract of Returns from County and Borough Asylums, Registered Hospitals, and Licensed Houses, and the Royal Hospital of Bethlehem. March,* 1852.

| Circumstances. | | Indictable Offences. | | | | | | | | | Other Cases. | | | | | | Grand Total. | | |
|---|---|---|---|---|---|---|---|---|---|---|---|---|---|---|---|---|---|---|---|
| | | Felonies. | | | | | | Misdemeanors. | | | Committed for want of Sureties, or as Insane Persons suspected of Criminal Intentions. | | | Summarily convicted for Vagrancy or Wilful Damage, and become Insane. | | | | | |
| | | Against Life. | | | Against Property and Persons. | | | | | | | | | | | | | | |
| | | M. | F. | Total. | M. | F. | Total. | M. | F. | Total. | M. | F. | Total. | M. | F. | Total. | M. | F. | Total. |
| Tried . . . | Acquitted on ground of Insanity . . . . | 80 | 26 | 106 | 25 | 3 | 28 | 11 | 2 | 13 | — | — | — | — | — | — | — | — | — |
| | Convicted and sentenced, and become Insane . | 9 | 1 | 10 | 103 | 18 | 121 | 20 | 1 | 21 | — | — | — | — | — | — | — | — | — |
| Not yet tried | Found Insane on arraignment . . . . . Committed for trial, and become Insane . . | 15 | 7 | 22 | 35 | 4 | 39 | 4 | 2 | 6 | — | — | — | — | — | — | — | — | — |
| Totals . . . . . | | 104 | 34 | 138 | 163 | 25 | 188 | 35 | 5 | 40 | 36 | 7 | 43 | 22 | 8 | 30 | 360 | 79 | 439 |

